# Supplementary material for: A generic blood banking and transfusion process-oriented architecture for virtual organizations
Source: PLoS One. 2024 Jun 5;19(6):e0303970. doi: 10.1371/journal.pone.0303970 (PMC11152302; doi:10.1371/journal.pone.0303970)
Supplement: S1 Appendix — (DOCX) [file pone.0303970.s001.docx]

**S1_Appendix: List of Stakeholders title and number who responded to the research questionnaire**

| **Stakeholder title** | **Number of respondents** |
| --- | --- |
| Medical Lab Manager | 1 |
| Technical Lab Manager | 1 |
| Lab Technician | 2 |
| Hematologist | 2 |
| Physician | 2 |
| Registered Nurse | 2 |
| Quality Expert | 1 |
| Biowaste Disposal Provider | 1 |
| Water, Electricity Provider | 1 |
| Provision Service | 1 |
| Blood Donor | 1 |
| Cancer Patient | 1 |
| Central Temperature control Unit | 1 |
| Ethical Experts | 1 |
| Social Worker | 1 |
| Funding responsible | 1 |
