# Supplementary material for: A generic blood banking and transfusion process-oriented architecture for virtual organizations
Source: PLoS One. 2024 Jun 5;19(6):e0303970. doi: 10.1371/journal.pone.0303970 (PMC11152302; doi:10.1371/journal.pone.0303970)
Supplement: S2 Appendix — (DOCX) [file pone.0303970.s002.docx]

S2_Appendix: The research Questionnaire form

Research Questionnaire

This questionnaire is a requirement for the completion of the dissertation of the MSc in Cancer Care Informatics that is jointly run between King Hussein Cancer Center and The University of Jordan. The research aims to (from the acceptance letter)

In order in a accomplish this aim, a generalized process architecture for blood transfusion ought to be developed and therefore a questionnaire has been designed to elicit the candidate essential business entities (CEBEs) that are associated with blood transfusion as a virtual organisation. This questionnaire embodies 14 adapted questions following the Riva business process architecture modelling method in relation to heuristics that can be utilised to guide domain experts in proposing CEBEs for blood transfusion as a virtual organisation. Riva is a method for building object-based business process architectures. A business process architecture (BPA) is a blueprint of organisational processes, their types and dynamic relationships between them. Amongst the early Riva steps for constructing a Riva process architecture is the elicitation of essential business entities (EBEs), which later become as the Candiate EBEs (CEBEs) for the organisation in stud. An EBE is a thing or an entity that is essentially related to the business domain of interest such as blood transfusion in this research. Collectively, EBEs characterise the business of the organisation that the BPA relates to.

This questionnaire is voluntary and you have the right to withdraw at any time without any obligations. We hope you will fill it out, your responses will be confidential and will not release to any third parties.^1^

| **Part 1:**  **Personal information about the respondent:** | |
| --- | --- |
| 1. | Date _______________________ |
| 2. | Full name _______________________ |
| 3. | Institution affiliation _______________________ |
| 4. | Job _______________________ |
| 5. | Signature _______________________  Ould, M., 2018. Bussiness Process Management A Rigorous Approach. |

**Part 2:**

Below are the 14 Riva method adapted heuristics to assist domain stakeholders to identify CEBEs for a generalised blood transfusion virtual organisation. The following are examples are of EBEs that you may find them useful to orient your journey towards what an EBE example is:

1. Pharmaceutical R&D Company: Drug Compound, Clinical Trial, Assay, and Batch of Raw compound.^3^
2. Cancer Care: Cancer Detection Report and Pathological Investigation.

| **Adapted Riva Heuristics**  **To identify CEBEs** | | **Proposed CEBEs** | **Score of the degree of confidence with CEBE**  **(1-5)** | **Is this heuristic clear and informative?**  **(Yes / No)** | **Do you have a suggestion to modify or rephrase this heuristic?** |
| --- | --- | --- | --- | --- | --- |
| 1. | What do blood transfusion organisations produce? |  |  |  |  |
| 2. | What do blood transfusion organizations provide externally? |  |  |  |  |
| ^2,3^  Ould, M., 2018. *Business Process Management A Rigorous Approach*.  ^4^   Odeh, M., Green, S., Kossmann, M., Tbaishat, D., Tbakhi, A. and Mansour\, A., 2018. Evaluating the Riva Business Process Architecture Identification Method and its Constituent Heuristics through their Application to a Cancer Care Organisation. In: *2018 1st International Conference on Cancer Care Informatics (CCI)*. Amman. | | | | | |
|  | **Modified Riva Heuristics**  **To identify CEBEs** | **Identified CEBEs** | **Score the degree of confidence with CEBE**  **(1-5)** | **Does this heuristic is clear?**  **(Yes / No)** | **What is your suggestion to rephrase this heuristic?** |
| 3. | What provision lines do we have for blood transfusion? |  |  |  |  |
| 4. | What services do blood transfusion organisations offer? |  |  |  |  |
| 5. | What service lines do blood transfusion organisation have? |  |  |  |  |
| 6. | What things blood transfusion organisations cannot simply get away from? |  |  |  |  |
| 7. | Who are the external beneficiaries of blood transfusion? |  |  |  |  |
| 8. | Who are the internal beneficiaries of blood transfusion?  **Modified Riva Heuristics**  **To identify CEBEs** | **Identified CEBEs** | **Score the degree of confidence with CEBE**  **(1-5)** | **Does this heuristic is clear?**  **(Yes / No)** | **What is your suggestion to rephrase this heuristic?** |
| 9. | Are there things that our external and internal beneficiaries have, or want, or do, that might be EBEs for blood transfusion? |  |  |  |  |
| 10. | What things do blood transfusion organisations that should have in common and things that are uncommon? |  |  |  |  |
| 11. | What kind of things do blood donation organisation deal with day in and day out? |  |  |  |  |
| 12. | What events in the outside of blood transfusion organisations that need to be responded to?  **Modified Riva Heuristics**  **To identify CEBEs** | **Identified CEBEs** | **Score the degree of confidence with CEBE**  **(1-5)** | **Does this heuristic is clear?**  **(Yes / No)** | **What is your suggestion to rephrase this heuristic?** |
| 13. | What related entities should be considered in the corporate data model of blood transfusion organisations? |  |  |  |  |
| 14. | What things do blood transfusion information systems should keep information about when interoperating with other corporate information systems? |  |  |  |  |
