# Supplementary material for: A generic blood banking and transfusion process-oriented architecture for virtual organizations
Source: PLoS One. 2024 Jun 5;19(6):e0303970. doi: 10.1371/journal.pone.0303970 (PMC11152302; doi:10.1371/journal.pone.0303970)
Supplement: Attachment — Submitted filename: plos.docx [file pone.0303970.s003.docx]

1. In methods and design ,authors stated the involvement of 20 key stakeholders. Who were the stakeholders and what was the criteria for selection of them?
2. no clarity of response is earmarked in column four of table 2 about availability of process at KAUH
